# Operant Conditioning in Honey Bees (*Apis mellifera* L.): The Cap Pushing Response

**DOI:** 10.1371/journal.pone.0162347

**Published:** 2016-09-14

**Authors:** Charles I. Abramson, Christopher W. Dinges, Harrington Wells

**Affiliations:** 1 Laboratory of Comparative Psychology and Behavioral Biology, Oklahoma State University, Stillwater, Oklahoma, United States of America; 2 Department of Biology, University of Tulsa, Tulsa, Oklahoma, United States of America; University of California San Diego, UNITED STATES

## Abstract

The honey bee has been an important model organism for studying learning and memory. More recently, the honey bee has become a valuable model to understand perception and cognition. However, the techniques used to explore psychological phenomena in honey bees have been limited to only a few primary methodologies such as the proboscis extension reflex, sting extension reflex, and free flying target discrimination-tasks. Methods to explore operant conditioning in bees and other invertebrates are not as varied as with vertebrates. This may be due to the availability of a suitable response requirement. In this manuscript we offer a new method to explore operant conditioning in honey bees: the cap pushing response (CPR). We used the CPR to test for difference in learning curves between novel auto-shaping and more traditional explicit-shaping. The CPR protocol requires bees to exhibit a novel behavior by pushing a cap to uncover a food source. Using the CPR protocol we tested the effects of both explicit-shaping and auto-shaping techniques on operant conditioning. The goodness of fit and lack of fit of these data to the Rescorla-Wagner learning-curve model, widely used in classical conditioning studies, was tested. The model fit well to both control and explicit-shaping results, but only for a limited number of trials. Learning ceased rather than continuing to asymptotically approach the physiological most accurate possible. Rate of learning differed between shaped and control bee treatments. Learning rate was about 3 times faster for shaped bees, but for all measures of proficiency control and shaped bees reached the same level. Auto-shaped bees showed one-trial learning rather than the asymptotic approach to a maximal efficiency. However, in terms of return-time, the auto-shaped bees’ learning did not carry over to the covered-well test treatments.

## Introduction

Operant conditioning as originally envisioned by B. F. Skinner is characterized by goal-directed motor manipulation of the environment [1]. This manipulation was achieved by substituting an arbitrary response such as a lever press for the locomotive response associated with such commonly used apparatus as the runway, running wheel, and maze. The role of species typical behavior was minimize by using an arbitrary response in the expectation that it would help stimulate a “functional analysis” of behavior. This arbitrary response was created by a process known as shaping (or “response differentiation by successive approximations”) [2]. Shaping consists of the use of reward and non-reward to reinforce increasingly accurate responses by a subject, leading to a final, arbitrary, response the organism would not otherwise produce. The creation of this arbitrary behavior by the use of shaping has become one of the defining characteristics of operant behavior and, in our view, one of the most important behavioral principles in psychology. As one of the most widely employed treatment techniques used in applied behavior analysis, shaping is most commonly used in applied treatments for individuals with autism spectrum disorder [3], and to aid stroke patients in overcoming learned non-use of limbs [4–6]. Despite its popularity in applied fields and vertebrate research, shaping is rarely investigated in invertebrates, an area potentially ripe for investigation of the neuronal basis of learning and memory. The present study uses a newly developed shaping protocol to examine operant conditioning in an invertebrate, the honey bee.

Honey bees represent an important insect model in the study of learning and memory, and two techniques have dominated this research: PER and SER [7, 8]. The proboscis extension reflex (PER) pairs a scent with an unconditioned stimulus to elicit an appetitive associative learning response. In contrast, the sting extension reflex (SER) pairs a scent with an electric shock to illicit an aversive associative learning response.

The PER technique in immobilized honey bees produces a true associative learning situation [9]. Although the first published use of a proboscis extension reflex technique in honey bees was 70 years ago [10], only after refinements making experimental results highly repeatable and applicable to psychology learning models [11–14] has the protocol become an attractive mean for studying appetitive classical conditioning roles in a wide variety of learning and memory scenarios [7]. PER methodology has elucidated many cognitive similarities between honey bees and vertebrates [15, 16], and is proving to be an important tool for discovering the underlying cellular and molecular processes involved in classical conditioning [17–22]. PER experiments show that honey bees may accomplish the same task as vertebrates but cognitively do so in a different manner. A case in point is the PER demonstrated inability of honey bees to use the removal of an odor stimulus as a conditioning cue [23].

In contrast to the PER modality, the sting extension reflex is an aversive, classical conditioning experimental design that also utilizes a harnessed bee [24]. Like the PER technique, the SER protocol has also been an important tool in studying the molecular basis of classical conditioning. It is clear that aversive long-term memory involves protein synthesis [25], and that the ecdysone/dopamine signaling pathway is involved in aversive classical conditioning [26]. The SER methodology actually has its roots in electro-shock aversive learning of free flying honey bees [27, 28], and has also been successfully used in shuttle box experiments [28–31]. The shuttle box design allows a broader range of learning models to be tested than simple SER, and in fact showed that the SER technique (harnessed bee) actually presented an aversive rather than positive ‘attack’ conditioning situation [29]. The shuttle box experimental design also opens a new leaning domain for study: operant conditioning.

Operant conditioning centers on learning from the consequences of behavioral choices [32], and is less well studied in invertebrates than is classical conditioning when compared to the vertebrate counterparts [33, 34]. Thus far, accounts of operant conditioning in invertebrates are limited to behaviors such as discrimination in Y- or T-mazes [35, 36], place preference in shuttle boxes [31, 37], and response rate change operant chambers [34, 38–40]. Specific genetic mutants in *Drosophila* affect classical conditioning, and it is interesting that some of those decrease classical learning mutants have no effect on operant learning [41]. Thus, even on the most basic level there are fundamental differences between classical and operant learning.

Like classical conditioning, operant conditioning has been a dominant element in the analysis of learning across the animal kingdom [42], not only for the insight provided in basic animal cognition but also as a means to explore how environmental factors impacts behavioral responses. Illustrative examples of the latter include alcohol in primates [43], pesticide ingestion by insects [9, 44], and the broad field of ‘cognitive ecology’ [45]. Various forms of operant conditioning provide the determinants for advanced cognitive processes [46, 47] and capacities for conceptual learning even in organisms with neuronal systems as relatively simple as the honey bee [48]. *Drosophila*, *Aplysia*, and *Lymnaea* have been important invertebrate models that have provided insights into the cellular and molecular basis of operant learning beyond what can be gleaned from their vertebrate counterparts [33], and *Apis mellifera* is now proving as valuable due to its rich behavioral repertoire associated with foraging and social interactions [46, 49, 50].

In this paper we present a new modality, the cap pushing response (CPR), for exploring operant behavior that lends itself to studies involving behavioral shaping of honey bees. The CPR protocol requires bees to both exhibit a truly novel behavior and in doing so operate ‘manipulata’ in a process that mirrors the lever-pushing protocols used by Skinner with vertebrates such as rats and pigeons [32]. Using the CPR protocol we test for the effect of explicit- versus auto-shaping on learning curves.

## Materials and Methods

### Experimental Design

Experiments were conducted spring of 2015 at the Department of Entomology of the Pontificia Universidad Católica de Valparaíso, in Quillota Chile. Honey bees (*Apis mellifera* L.) housed in a laboratory hive at the Pontificia Universidad Católica de Valparaíso were trained to a feeding platform 18 m from the hive following the methods of Abramson (1990) [51]. The training platform contained a 59 mm diameter gray plastic disk with a 6 ml drop of 50% (w/v) sucrose solution in the center as reward. Free-flying forager honey bees making repeated trips to the foraging platform were used in the experiments. One bee was tested at a time, with N = 55 bees in total used in experiments. All other bees were captured and removed from the location.

The experiment was initiated when the test subject returned to the hive. The gray plastic disk was removed and replaced by an experimental target that consisted of a clear 88 mm diameter plastic disk. The disk had a red 50 mm diameter circle painted in the center of the disk on the underside. In the center was a feeding well 10 mm in diameter and 6 mm deep capable of holding 20 ml of 50% sucrose solution (enough for three visits from one bee). A hollow plastic cap, measuring 12 mm in diameter, 10 mm in height, and weighing 0.12g, was used to cover the feeding well in some trials of the experiment. The cap was hollow, open only on the bottom. To control for odor cues, feeding disk and cap were changed between and within experimental trials. The feeding disks and caps were washed, triple rinsed with distilled water, and air dried between uses.

Three different experiments were performed: 1) Control, 2) Explicit-Shaping, and 3) Auto-Shaping. Each bee experienced only one of the three experimental protocols (N = 20 bees Control, N = 20 bees Explicit-Shaping, N = 15 bees Auto-Shaping). The first two experiments were run initially, and the results of those experiments led to the Auto-Shaping experiment. To control for calendar variables, bees from the Control and Explicit-Shaping groups were run intermixed, but just one bee at a time. Each run of an experiment consisted of giving a bee 20 sequential, uninterrupted trials, where each trial represented a return trip from the hive. Recorded on each trial in both experiments were: 1) return time, 2) number of cover pushes per-trial, and 3) latency to push cover from time of landing on the disk.

The Auto-Shaping experiment was subsequently performed. Each run of an experiment consisted of giving a bee 20 sequential, uninterrupted trials, where each trial represented a return trip from the hive. Recorded on each trial in both experiments were: 1) return time, 2) number of cover pushes per-trial, and 3) latency to push cover from time of landing on the disk.

### Control group

Each bee was given 20 trials. The first 5 trials (*baseline*) utilized a disk with an uncovered feeding well. Beginning on trial 6, bees encountered the feeding well fully covered by the cap on each return trip (each trial). If a Control group bee failed to push the cap to gain access to the reward within 10 minutes, the experiment was terminated for that bee since our previous work shows that bees will abandon a situation if they are not rewarded within 10 minutes [28, 40]. If the Control group bee pushed the target, the experiment continued for an additional 15 trials for a total of 20 trials.

### Explicit-Shaping group

Each bee was given 20 trials. Like the Control group, the first 5 trials (*baseline*) utilized a disk with an uncovered feeding well. During trials 6–10 bees experienced a behavioral shaping regiment to push the cap. On trial 6, half of the feeding well was covered by the cap (5mm). On trials 7, 8, 9 and 10 the cap progressively covered 1 mm more of the feeding well. The purpose of the shaping phase was to give the bee explicit experience in pushing the cover. Trials 11–20 matched the Control group where the cap completely covered the feeding well, and to gain access to the sucrose reward the bee had to push the cap to uncover the reward.

### Auto-Shaping Experiment

Each bee was given 20 trials. Like the Control and Explicit-Shaping groups, the first 5 trials (*baseline*) utilized a disk with an uncovered feeding well. During trials 6–10 bees experienced a situation made to elicit behavioral auto-shaping to push the cap. During these 5 trials bees were given the cap inverted, covering all but 0.5mm of the feeding well. This allowed bees to access the reward via proboscis extension without moving the cap. Trials 11–20 matched that of the Control and Explicit-Shaping groups where the cap covered the feeding well completely; to gain access to the sucrose reward the bee had to push the cap to uncover the feeding well.

### Statistical Analysis of Data

Data were fitted to the Rescorla-Wagner model of learning curves. The model predicts that the rate of learning is proportional to the difference between the current ability to solve the problem (measure of ability) and the physiological limit possible. The model is semi-log in nature and can be expressed in the form *ln*(*c*_*n*_ *− c*_*∞*_) *= −an + ln*(*c*_0_ *− c*_*∞*_) where *c*_*n*_ is the trial accuracy, *c*_∞_ is the physiologically possible best accuracy, *c*_*o*_ is the accuracy on the initial trial, *a* is the learning-rate parameter, and *n* is the trial number [52]. The model fit is thus a line with y = *ln*(*c*_*n*_ *− c*_*∞*_) and x = *n* where *c*_*n*_ is the current measure of learning and ***c***_∞_ is a constant. We used 3 separate measure of learning, and analyze each separately. Thus, *c*_*n*_ is ‘return-time’, ‘latency-time’, or ‘pushes’ depending upon the analysis.

Each treatment of each experiment experienced by a cohort of bees was fit to the model and statistically analyzed via regression analysis using the SAS program JMP [53, 54]. For Return-Times we used *c*_*∞*_ = 180 sec which was 9 sec faster than observed for any bee in any treatment for Control, Explicit-Shaping, or Auto-Shaping group subjects. For Latency-Times we used *c*_*∞*_ = 1 sec which was 1.2 sec faster than observed for any bee in any treatment for Control, Explicit-Shaping, or Auto-Shaping group subjects. When dealing with the number of cap pushes, the results were integer values greater than or equal to 1 and many bees were able to move the cover in a single push. Thus, we used *c*_*∞*_ = 0.9 to resolve this issue (*i*.*e*. *ln*(0)) and were able to analyze the entire data set.

## Results

Our results, presented in detail in the following sections, demonstrate that honey bees are capable of developing the novel CPR tactic to access a concealed food source, both with and without explicit shaping. Experience is critical to rapid mastery of this strategy. The results of the Control and Explicit-Shaping experiment led to the Auto-Shaping experiment presented below.

### A. Control and Explicit-Shaping Experiments

Bees in the Control group were given two treatments: ‘*baseline*’ where the feeding well was uncovered (trials 1 through 5), and ‘*covered*’ where the feeding well was covered by a moveable cap (trials 6 through 20). Bees in the Explicit-Shaping group were given 3 treatments: ‘*baseline*’ where the feeding well was uncovered (trials 1 through 5), ‘*shaping*’ where the feeding well was progressively covered by a movable cap over multiple trial (trials 6 through 10), and ‘*covered*’ where the feeding well was completely covered by a moveable cap (trials 11 through 20).

#### Return times

**Fig 1:**
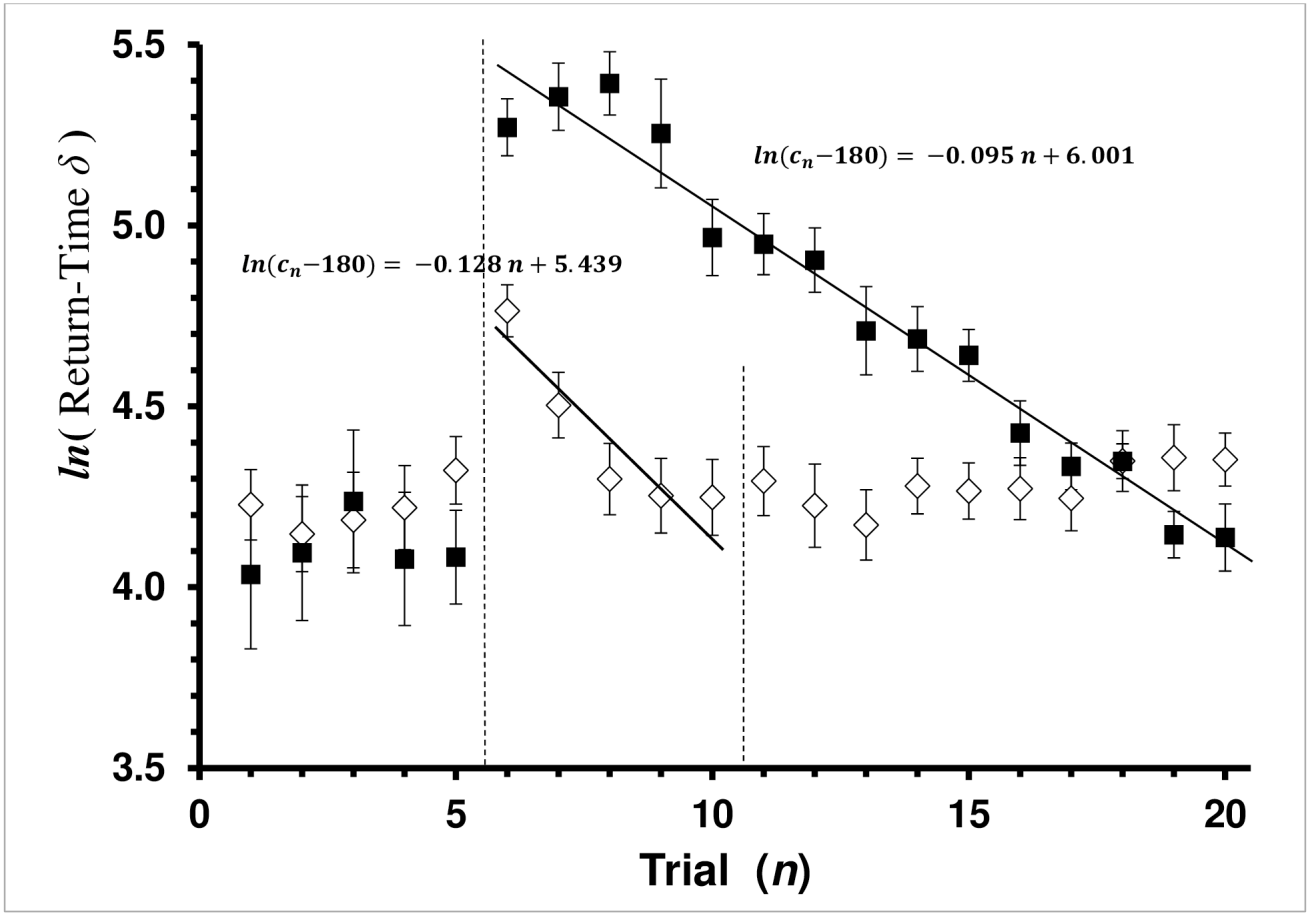
Average Return-Time *δ* by Trial for Control and Explicit-Shaping Groups. Return-Time *δ* = (*c*_*n*_−*c*_c∞_) is the difference between the current-trial (*c*_*n*_) and physiologically shortest possible return time (*c*_c∞_ = 180 sec in this study). Average return-time *δ* with standard error bars is presented. Each square, ■, is the mean of a trial for the Control group. Each diamond, ◊, is the mean of a trial for the Explicit-Shaping Experiment group. Vertical dashed lines mark treatment boundaries. Trials 1–5 are the *baseline* treatment for both Control and Explicit-Shaping bees. Trials 6–10 are the *shaping* treatment for the Explicit Shaped group. Trials 11–20 are the *covered* treatment for the Explicit-Shaping bees, while trials 6–20 are the *covered* treatment for the Control bees. Control bees only experienced two treatments (*baseline* and *covered*) while the Explicit-Shaping group of bees experienced three treatments (*baseline*, *shaping*, and *covered*). Regression lines shown are the least-square fit for the Rescorla-Wagner learning model, (*c*_*n*_ *− c*_*∞*_) *= −a n + ln*(*c*_0_ *− c*_*∞*_).

The Control group consisted of 20 bees. Five of those 20 bees failed to return on trial 6, abandoning the foraging site upon experiencing the cap-covered well. The remaining 15 bees completed all 20 trials. The regression coefficient was not significant in the *baseline* treatment for either the 5 bees that abandoned the site (ANOVA: F_1,23_ = 0.0315, *P =* 0.8606) or the 15 bees that completed both the *baseline* and *covered* treatments, which together encompassed 20 trials (ANOVA: F_1,73_ = 0.0845, *P =* 0.7721). Further, the mean return time did not differ significantly between the 5 bees that abandoned the site and the 15 bees that complete all treatments during the *baseline* period (t test: T_98_ = 0.01498. *P =* 0.9881). Combining the *baseline* data from these two sets of bees (5 bees that abandoned the site and 15 bees that completed all treatments), the average return-time fitting the mean over time was 240.7 sec (Fig 1).

Bees in the Control group that completed the 20 trials initially experienced difficulty reaching the sucrose when the well was covered, but progressively became more efficient at doing so. The regression coefficient was significant (ANOVA: F_1,223_ = 282.9589, *P<*0.0001), and learning appeared to occur at the rate predicted by the model across the *covered* trials, trials 6 through 20 (Fig 1). Return time diminished by half in 7.3 trials (model half-life). The Lack-of-Fit test for the model was not significant (ANOVA: F_13,210_ = 0.7683, *P =* 0.6931).

Bees in the Explicit-Shaping group all completed the 20 trials (20 bees). This represented a significant difference from the Control group (*X*_*1*_^2^ = 5.7143, *P =* 0.0168). However, like the Control group bees, bees in the Explicit-Shaping group did not have a significant regression coefficient for the *baseline* trials (ANOVA: F_1,198_ = 0.5632, *P =* 0.4548). Bee average return-time fitting the mean over time was 248.1 sec (Fig 1).

Like the Control group, bees in the Explicit-Shaping group initially experience difficulty reaching the reward when the cap was present in the *shaping* treatment even though it did not completely cover the well, but the bees rapidly mastered the task as the well become progressively more obscure. The regression coefficient was significant (ANOVA: F_1,98_ = 17.3725, *P<*0.0001), and the Lack-of-Fit test for the model was not significant (ANOVA: F_3,95_ = 1.1631, *P =* 0.3280). The regression-coefficient of the Explicit-Shaping group over the *shaping* trials was greater (negative) than the Control group regression-coefficient over the *covered* 15 trials where learning occurred (F test: F_1,321_ = 22.8587, *P<*0.0001). Learning for Explicit-Shaping group bees appears complete by the end trial in the *shaping* treatment (Fig 1), and the regression coefficient was not significant in the *covered* treatment for these bees (ANOVA: F_8,190_ = 1.8836, *P =* 0.1715).

#### Latency to hit cover

**Fig 2:**
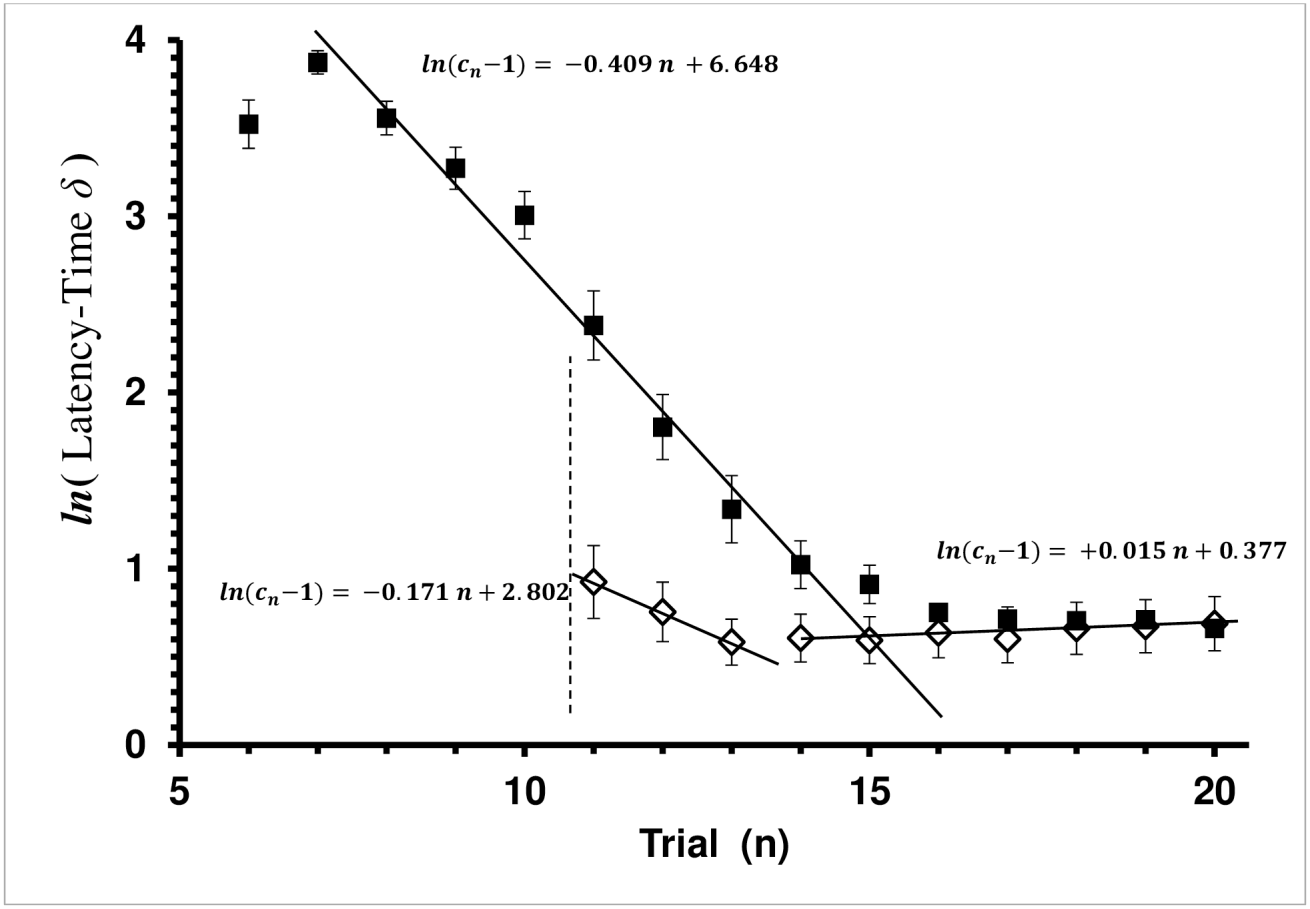
Average Latency-Time *δ* by Trial for Control and Explicit-Shaping Groups in the Covered Treatment. Latency-Time *δ* = (*c*_*n*_−*c*_c∞_) is the difference between the current-trial (*c*_*n*_) and physiologically shortest possible latency time (*c*_c∞_ = 1 sec in this study). Average latency-time *δ* with standard error bars is presented. Each square, ■, is the mean of a trial for the Control group. Each diamond, ◊, is the mean of a trial for the Explicit-Shaping Experiment group. Vertical dashed lines mark treatment boundaries. Trials 6–10 are the *shaping* treatment for the Explicit Shaped group. Trials 11–20 are the *covered* treatment for the Explicit-Shaping bees, while trials 6–20 are the *covered* treatment for the Control bees. Trials 1–5 (not shown) are the *baseline* treatment for both Control and Explicit-Shaping bees. Since there was no cap covering the feeding well, there was no latency-time between landing and pushing the cap. Regression lines shown are the least-square fit for the Rescorla-Wagner learning model, (*c*_*n*_ *− c*_*∞*_) *= −a n + ln*(*c*_0_ *− c*_*∞*_).

The Control group consisted of 15 returning bees, which experienced 15 consecutive trials (trials 6–20, since trials 1–5 were the *baseline* treatment). The regressions was significant (*P<*0.0001), but also was the Lack-of-Fit (*P<*0.0001). The areas where the data did not fit the Rescorla-Wagner model can be seen (Fig 2) to be the initial trial (trial 6) and the last five trials (trials 16–20). For trials 7 through 15 the regression coefficient was significant (ANOVA: F_1,133_ = 449.2533, *P<*0.0001), and the Lack-of-Fit test for the model was not significant (ANOVA: F_7,126_ = 1.2427, *P =* 0.2845). Latency-time diminished by half in 1.7 trials (model half-life). Learning stopped during the last five trials (trials 16–20); the regression coefficient was not significant (ANOVA: F_1,73_ = 1.4429, *P =* 0.2335). Bee average latency-time fitting the mean over time was 3.03 sec (Fig 2).

The Explicit-Shaping group (N = 20) experienced the *covered* treatment in trials 11–20, since the *baseline* treatment was trials 1–5 and the *shaping* treatment was trials 6–10. Like the Control group, the regression was significant (*P<*0.0045), but also was the Lack-of-Fit to the Rescorla-Wagner model (*P<*0.0001). Learning had stopped after the first 3 trials (Fig 2). The regression coefficient was significant for trials 11 through 13 (ANOVA: F_1,58_ = 23.4244, *P<*0.0001), and the Lack-of-Fit test for the model was not significant (ANOVA: F_1,57_ = 0.0003, *P =* 0.9857). As expected, learning to solve the problem had occurred in the *shaping* treatment, with the mean latency time in the first trial (trial 11) being only 3.60±0.16 sec (mean ± se) compared to 39.18±4.25 sec in the first trial (trial 6) of the Control group. Surprisingly, a slight increase in latency-time occurred in the Explicit-Shaping group over trials 14–20 (ANOVA: F_1,138_ = 5.3522, *P =* 0.0169), with a non-significant Lack-of-Fit (ANOVA: F_5,133_ = 0.3666, *P =* 0.8707).

Shaping led to a significant difference in task performance by trial 11 when compared to the Control group. On trial 11 the mean latency time differed between the two groups (t test: T_33_ = 4.9668, *P<*0.0001).

#### Number of cover pushes

**Fig 3:**
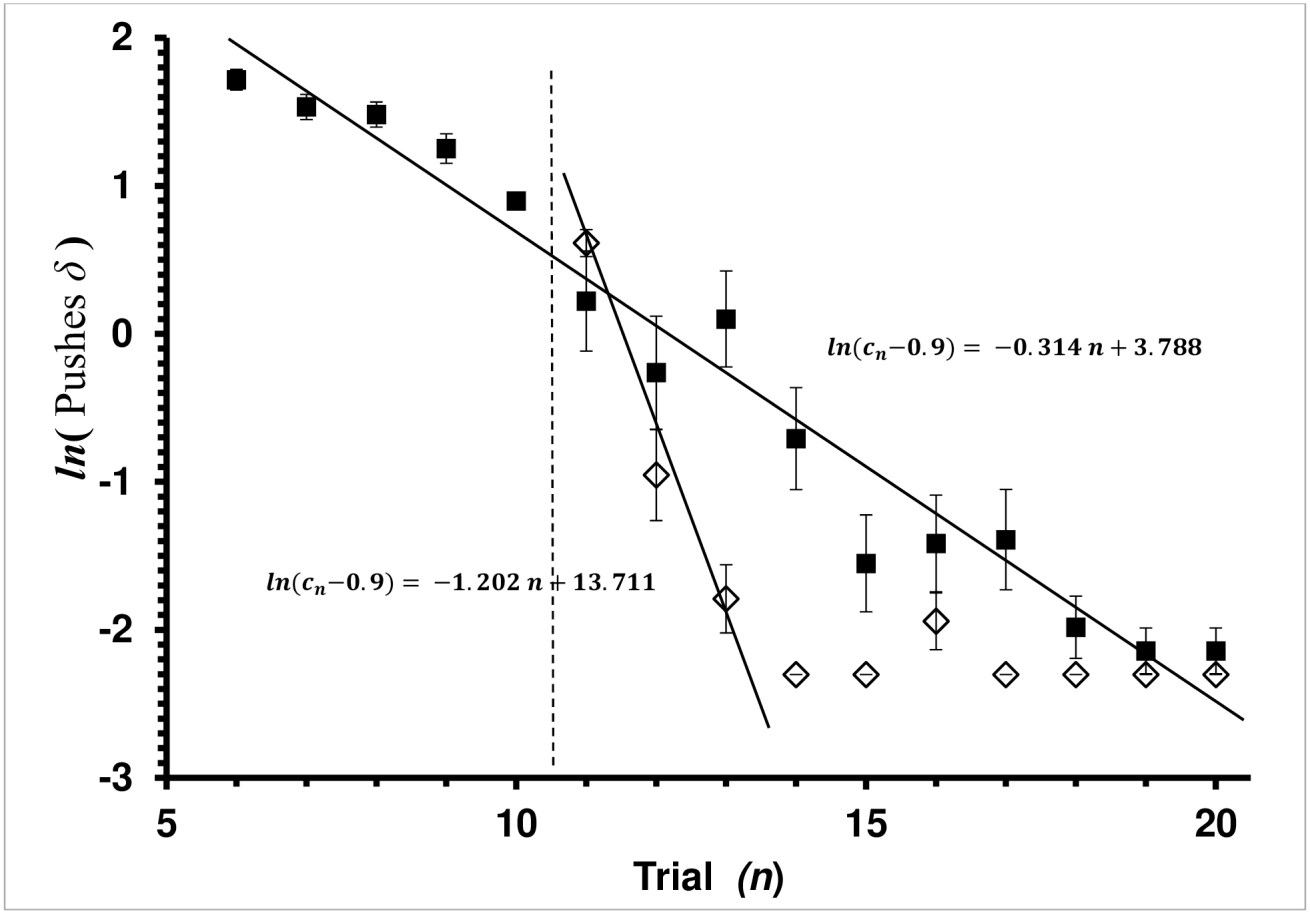
Average Pushes *δ* by Trial for Control and Explicit-Shaping Groups in the Covered Treatment. Pushes *δ* = (*c*_*n*_−*c*_c∞_) is the difference between the current-trial (*c*_*n*_) and physiologically least possible number of cap pushes (*c*_c∞_ = 0.9 used in this study—see Materials and Methods). Average cap Pushes *δ* with standard error bars is presented. Each square, ■, is the mean of a trial for the Control group. Each diamond, ◊, is the mean of a trial for the Explicit-Shaping Experiment group. Vertical dashed lines mark treatment boundaries. Trials 6–10 are the *shaping* treatment for the Explicit Shaped group. Trials 11–20 are the *covered* treatment for the Explicit-Shaping bees, while trials 6–20 are the *covered* treatment for the Control bees. Trials 1–5 (not shown) are the *baseline* treatment for both Control and Explicit-Shaping bees. Since there was no cap covering the feeding well, there was no cap pushes to gain access to the feeding well. Regression lines shown are the least-square fit for the Rescorla-Wagner learning model, *ln*(*c*_*n*_ *− c*_*∞*_) *= −a n + ln*(*c*_0_ *− c*_*∞*_).

The regession coefficient was significant for the Control group bees (ANOVA: F_1,223_ = 406.3820, *P<*0.0001) and the Lack-of-Fit was not significant (ANOVA: F_13,210_ = 1.2371, *P =* 0.2547) for trials 6 through 20. Thus, the Rescorla-Wagner model explained the learning curve for the Control group well over the entire *covered* set of trials (Fig 3). The number of pushes needed to reveal the feeding well diminished by half in 2.2 trials (model half-life).

In contrast, the regression was significant (ANOVA: F_1,198_ = 113.3754, *P<*0.0001) but the Lack-of-Fit was also highly significant (ANOVA: F_8,190_ = 22.9589, *P<*0.0001) for the Explicit-Shaping bees over the entire set of *covered* trials (trials 11–20). Bees became very proficient at moving the cover by trial 14. The regression was not significant when performed on the Explicit-Shaping group trials 14–20 (ANOVA: F_1,138_ = 0.7596, *P =* 0.3850). Bee average Number of Pushes fitting the mean over time was 1.0053 (Fig 3). For trials 11 to 13 where learning occurred in the Explicit-Shaping group, the regression was significant (ANOVA: F_1,58_ = 52.2006, *P<*0.0001) and the Lack-of-Fit was not significant (ANOVA: F_1,57_ = 1.6314, *P =* 0.2067).

The *shaping* treatment of the Explicit-Shaping group did not have a significant effect on learning when compared to the Control group which experienced the *covered* treatment over the same set of trials. On trial 11 the mean number of pushes did not differ between the two groups (t test: T_33_ = 1.2565, *P =* 0.2177), which was in contrast to the observation of Latency times. However, shaping did lead to an accelerated reduction in the number of pushes to move the cover off of the feeding well in the Explicit-Shaping group once the *covered* treatment began. The regression slopes were significantly different (ANOVA: F_1,284_ = 12.9603, *P =* 0.0004) with the Explicit-Shaping group having the greater negative slope.

### B. Auto-Shaping Experiment

Bees in the Auto-Shaping experiment were given 3 treatments: ‘*baseline*’ where the feeding well was uncovered (trials 1 through 5), ‘*auto-shaping*’ where the feeding well was covered by an inverted cap that allowed bees to access the reward via proboscis extension without moving the cap (trials 6 through 10), and ‘*covered*’ where the feeding well was completely covered by a moveable cap (trials 11 through 20).

#### Return times

**Fig 4:**
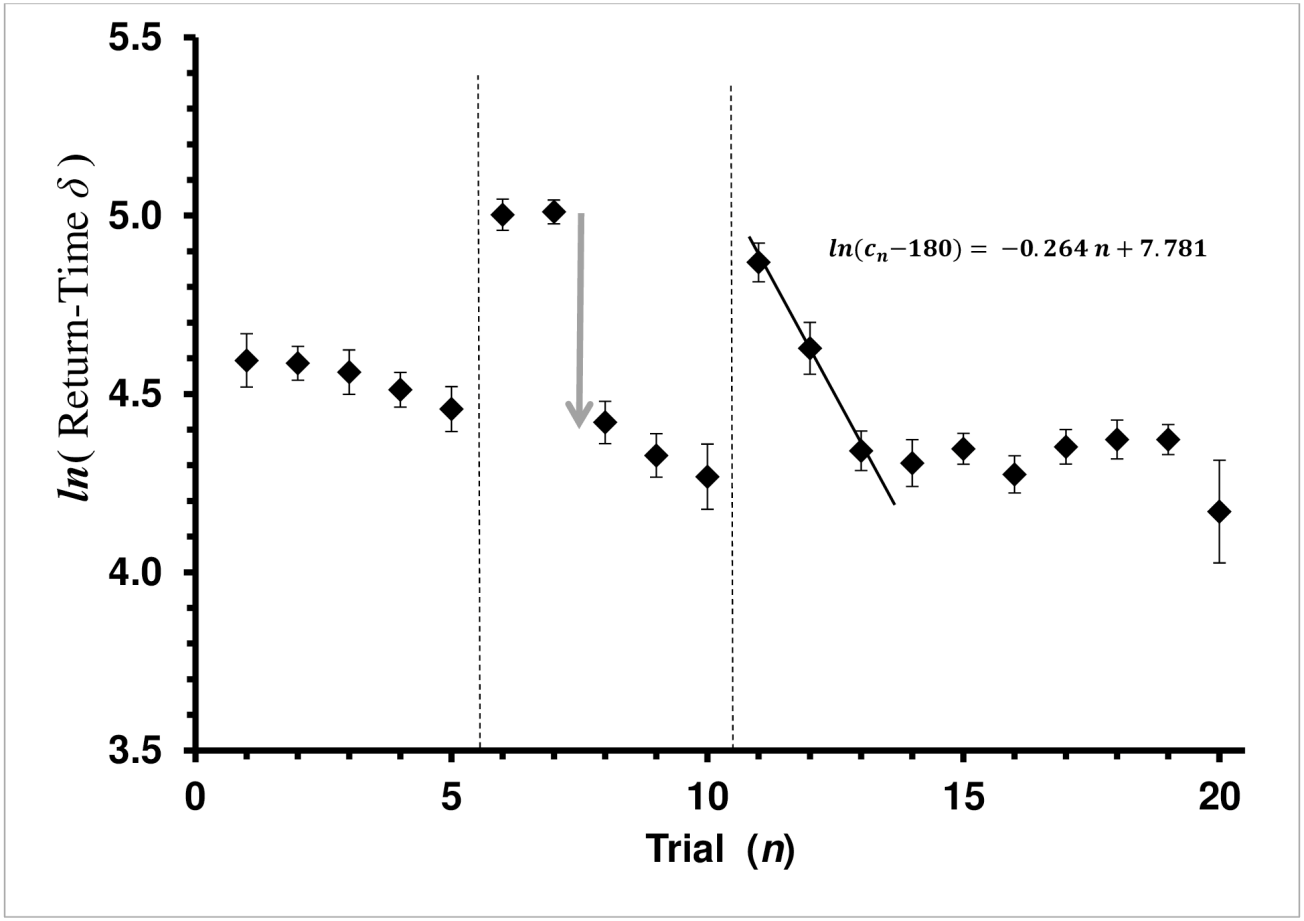
Average Return-Time *δ* by Trial for the Auto-Shaping Group in Shaping and Covered Treatments. Return-Time *δ* = (*c*_*n*_−*c*_c∞_) is the difference between the current-trial (*c*_*n*_) and physiologically shortest possible return time (*c*_c∞_ = 180 sec in this study). Average return-time *δ* with standard error bars is presented. Each diamond, ♦, is the mean of a trial for the Auto-Shaping Experiment group. Vertical dashed lines mark treatment boundaries. Trials 1–5 are the *baseline* treatment, trials 6–10 are the *auto*-*shaping* treatment, and trials 11–20 are the *covered* treatment for the Auto-Shaping bees. Regression lines shown are the least-square fit for the Rescorla-Wagner learning model, (*c*_*n*_ *− c*_*∞*_) *= −a n + ln*(*c*_0_ *− c*_*∞*_). Arrow pointing down represents one-trial leaning event, which does not fit the Rescorla-Wagner model.

Like the Explicit-Shaping group, bees in the Auto-Shaping experiment all completed the 20 trials (15 bees). Further, the regression coefficient for the *baseline* trials was not significant (ANOVA: F_1,73_ = 3.2370, *P =* 0.070). Bee average return-time fitting the mean over time was 273.9 sec (Fig 4).

The learning curve for the Auto-Shaping bees did not fit the Rescorla-Wagner model during the *shaping* treatment. Interestingly, the regression coefficient was significant (ANOVA: F_1,73_ = 92.7751, *P<*0.0001), as was the Lack-of-Fit test for the model (ANOVA: F_3,73_ = 6.9976, *P =* 0.0003). This is due to a one-trial learning event occurring midway through the shaping trials, rather than the Rescorla-Wagner asymptotic approach to the physiological best possible over the set of trials (Fig 4). Thus, return-time response of the Auto-Shaped group and Explicit-Shaped group were fundamentally different during the *shaping* treatment (trials 6–10). Explicit-Shaped bees became progressively more efficient over the series of trials in a Rescorla-Wagner learning curve manner, while the Auto-Shaped group reached its greatest proficiency in just one trial. Further, while learning from the explicit-shaping carried over to the *covered* treatment, the auto-shaping learning did not.

Unlike the Explicit-Shaping group, learning of the Auto-Shaped group in the *shaping* treatment did not carry over well to the *covered* treatment in the Auto-Shaping experiment (Fig 4). The regression coefficient for the final treatment (*cover*, trials 11–20) was significant (ANOVA: F_1,148_ = 31.0671, *P =* 0.0001) as was the Lack-of-Fit (ANOVA: F_8,140_ = 4.1883, *P =* 0.0002). The reason for the model lack of fit is that learning was complete by trial 13: bees had reached their best physiological performance (Fig 4). The regression for trials 11–13 was significant (ANOVA: F_1,42_ = 35.3336, *P =* 0.0001) with the Lack-of-Fit not significant (ANOVA: F_1,42_ = 0.0895, *P =* 0.7663). Further, the regression for trials 14–20 was not significant (ANOVA: F_1,103_ = 0.4299, *P =* 0.5135). The mean return time for trials 14–20 was 254.7 sec.

#### Latency to hit cover

**Fig 5:**
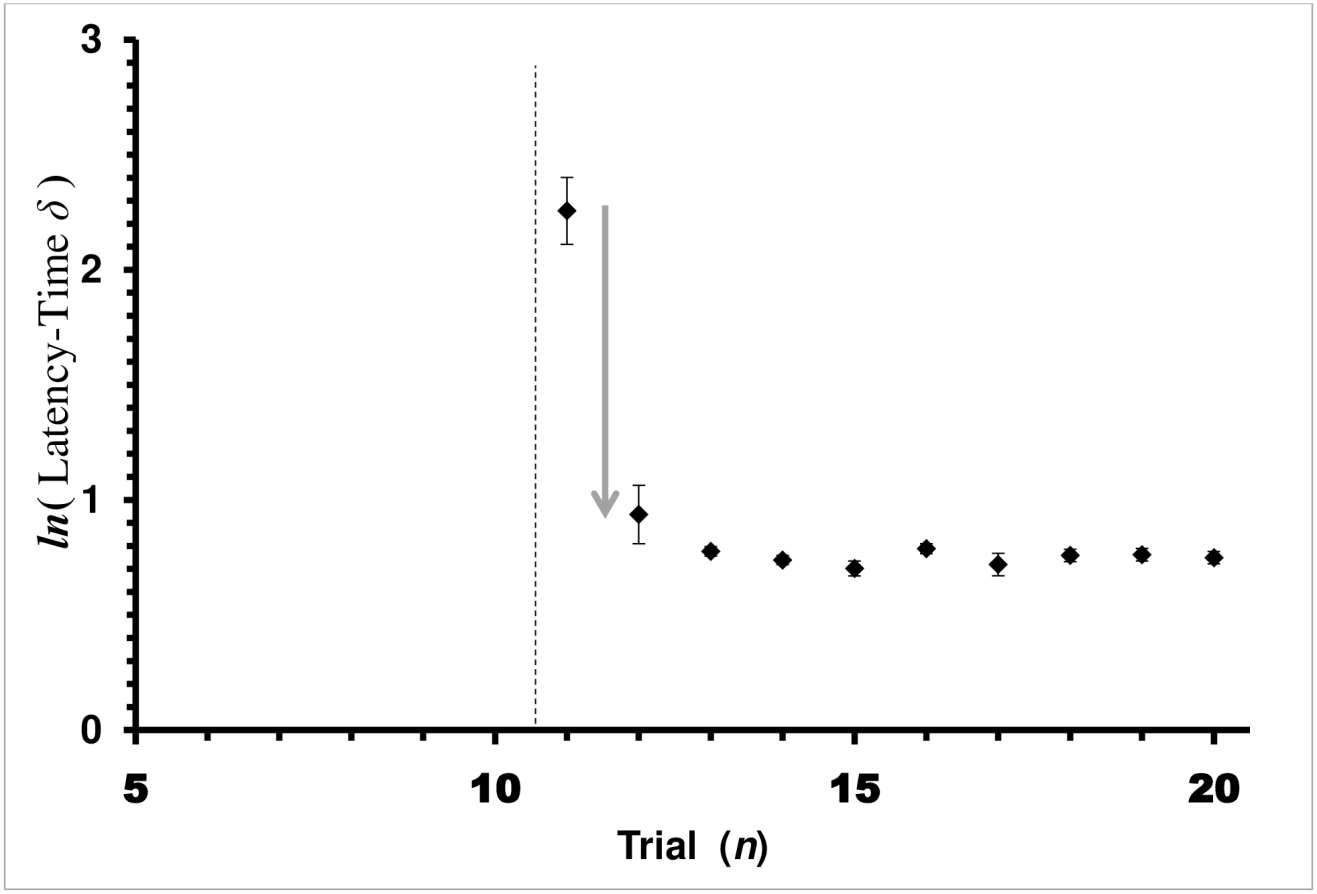
Average Latency-Time *δ* by Trial for the Auto-Shaping Group in Shaping and Covered Treatments. Latency-Time *δ* = (*c*_*n*_−*c*_c∞_) is the difference between the current-trial (*c*_*n*_) and physiologically shortest possible latency time (*c*_c∞_ = 1 sec in this study). Average latency-time *δ* with standard error bars is presented. Each diamond, ♦, is the mean of a trial for the Auto-Shaping Experiment group. Vertical dashed lines mark treatment boundaries. Trials 6–10 are the *auto*-*shaping* treatment (no points because bees did not have to push the cap to access reward), and trials 11–20 are the *covered* treatment for the Auto-Shaping bees. Trials 1–5 (not shown) are the *baseline* treatment. Since there was no cap covering the feeding well, there was no latency-time between landing and pushing the cap. Regression lines shown are the least-square fit for the Rescorla-Wagner learning model, *ln*(*c*_*n*_ *− c*_*∞*_) *= −a n + ln*(*c*_0_ *− c*_*∞*_). Arrow pointing down represents one-trial leaning event, which does not fit the Rescorla-Wagner model.

The results for Latency time have many similarities to the Return time. The learning curve for the Auto-Shaping bees did not fit the Rescorla-Wagner model. The regression coefficient was significant (ANOVA: F_1,148_ = 48.9478, *P<*0.0001), but so was the Lack-of-Fit test for the model (ANOVA: F_8,140_ = 35.7525, *P<*0.0001). Considering only trials 12–20, the regression was not significant (ANOVA: F_1,133_ = 3.2200, *P =* 0.0750). The mean latency time for trials 13–20 was 3.16 sec. Like the return times, latency time learning may best be described as a one-trial learning event (Fig 5) rather than the Rescorla-Wagner asymptotic approach to the physiological best possible over the set of trials. Notice that the latency times start at trial 11 at the same approximate value as the Control group on trial 11. However, the leaning model is completely different from trials 12–30. Like return-time, latency-time response of the Auto-Shaped group and Explicit-Shaped group were fundamentally different during the *shaping* treatment (trials 6–10). Explicit-Shaped bees became progressively more efficient over the series of trials in a Rescorla-Wagner learning curve manner, while the Auto-Shaped group reached its greatest proficiency in just one trial.

#### Number of cover pushes

**Fig 6:**
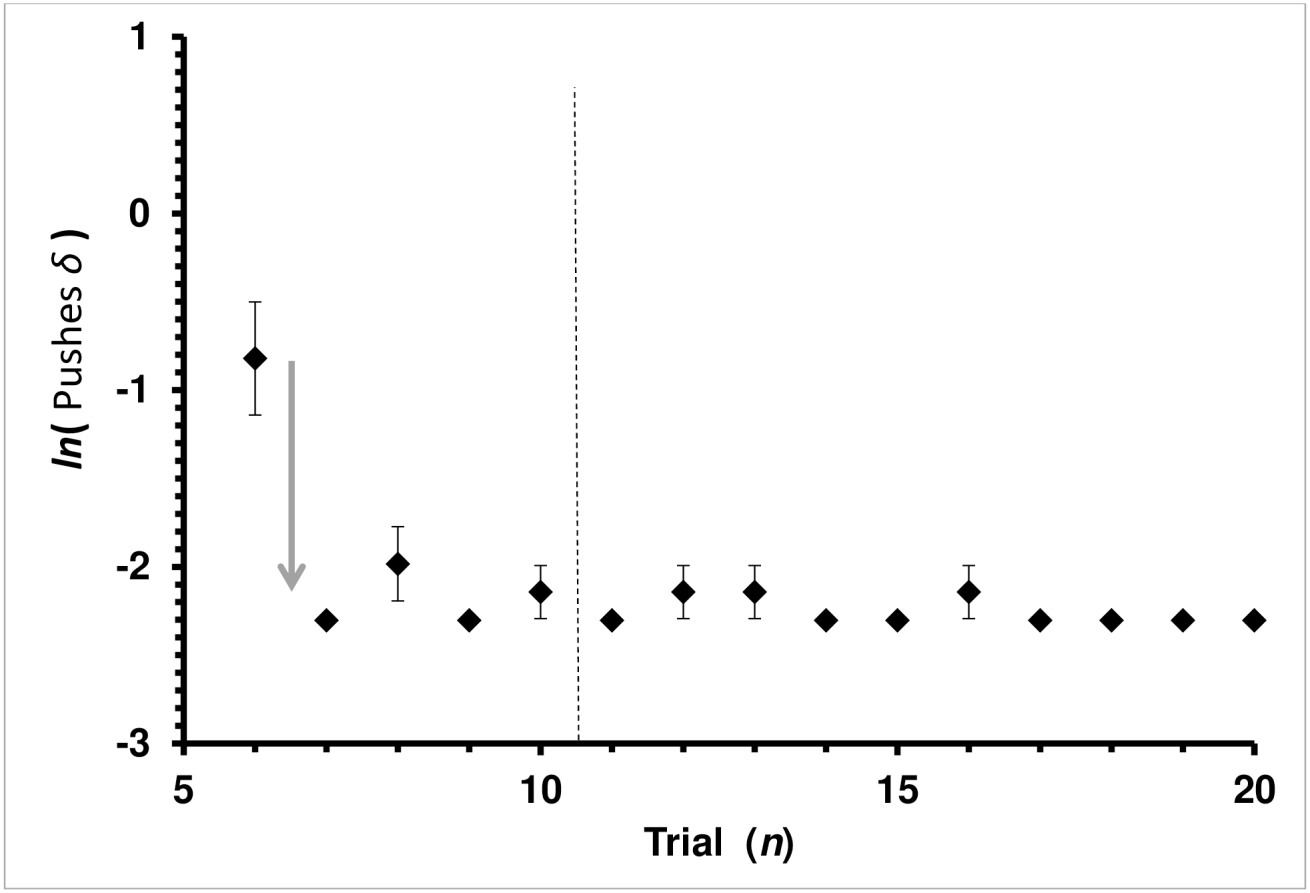
Average Pushes *δ* by Trial for the Auto-Shaping Group in the Covered Treatment. Pushes *δ* = (*c*_*n*_−*c*_c∞_) is the difference between the current-trial (*c*_*n*_) and physiologically least possible number of cap pushes (*c*_c∞_ = 0.9 used in this study—see Materials and Methods). Average cap Pushes *δ* with standard error bars is presented. Each diamond, ♦, is the mean of a trial for the Auto-Shaping group. Vertical dashed lines mark treatment boundaries. Trials 6–10 are the *auto*-*shaping* treatment while trials 11–20 are the *covered* treatment. Trials 1–5 (not shown) are the *baseline* treatment. Since there was no cap covering the feeding well, there was no cap pushes to gain access to the feeding well during the *baseline* treatment. Regression lines shown are the least-square fit for the Rescorla-Wagner learning model, *ln*(*c*_*n*_ *− c*_*∞*_) *= −a n + ln*(*c*_0_ *− c*_*∞*_). Arrow pointing down represents one-trial leaning event, which does not fit the Rescorla-Wagner model.

The results for Cover-Pushed are like that for Latency time. The learning curve for the Auto-Shaping bees did not fit the Rescorla-Wagner model. The regression coefficient was significant (ANOVA: F_1,223_ = 23.1550, *P<*0.0001), but so was the Lack-of-Fit test for the model (ANOVA: F_13,210_ = 6.6573, *P<*0.0001). Considering only trials 12–20, the regression was not significant (ANOVA: F_1,208_ = 2.0701, *P =* 0.1517), and neither was the Lack-of-Fit (ANOVA: F_12,196_ = 0.9097, *P =* 0.5383). The mean Cover-Pushes for trials 12–20 was 1.029 (SE 0.011). Like Latency times, Cover-Pushes learning may best be described as a one-trial learning event (Fig 6) rather than the Rescorla-Wagner asymptotic approach to the physiological best possible over the set of trials.

## Discussion

Existing literature focuses primarily on visual or odor discrimination; here we have provided the protocol foundation to investigate motor tasks in free flying foragers without the use of prohibitively expensive or complex apparatus. This protocol allows for a more varied and flexible response from the animal without sacrificing measurement accuracy. Further, the unambiguous cap pushing response can be readily applied to additional insect species, and allows for comparative analysis through a simple foraging task with universal applications.

Honey bees developed the novel CPR tactic to access a concealed food source. Experience is shown to more rapid mastery of this strategy. Control bees did not experience the *shaping* treatment, and 25% were never able to solve the problem of accessing the covered nectar well. Nevertheless, a majority (75%) of Control group bees (no *shaping* treatment) accessed the food by happenstance. These bees were eventually able to consistently and rapidly access the sucrose reward after repeated contact with the cap, but acquired proficiency much more slowly than bees explicitly shaped to do so (Explicit-Shaping group). These results were seen in all three measures of learning: return time, latency time, and cap pushes. The Rescorla-Wagner learning-curve model [52] fit the observations well for all three measures in both shaped and control bees. However, learning stopped after a set of trials rather than continuing endlessly as an asymptotic approach to the physiological leaning limit as predicted by the model.

The results of the Control and Explicit-Shaping experiments led to the Auto-Shaping experiment. In this experiment, the honey bees performed a self-shaping task without explicit shaping by the experimenter. Specifically, the well was covered but the inverted cap allowed a bee’s proboscis access to the reward through small cracks anywhere around the cap. Thus, access to the reward technically did not require pushing the cap but produced a situation where it was likely to occur. The *auto-shaping* trials resulted in a one-trial learning situation for return time rather than a Rescorla-Wagner learning curve. However, the *auto-shaping* learning did not carryover when the *covered* trials began, where a Rescorla-Wagner curve [52] was observed. Latency time was also a one-trial learning situation during *auto-shaping* trials, but interestingly carried over to the *covered* trials. The same result occurred for number of Cover-Pushes.

In this study, bees were trained to access a concealed food source by pushing a plastic cap. Bees successfully acquired this novel cap pushing response following either successive approximations (Explicit-Shaping group), or self-shaping through a task of intermediate difficulty (Auto-Shaping group). Replications of this experiment performed with heavier 3D printed caps (S1 Text) resulted in identical results for both Explicit-Shaping (S1 Video) and Auto-Shaping groups (S2 Video), but bees were unable to access the food source by happenstance in Control groups (S3 Video). Pilot manipulations have revealed interesting errors and patterns (S4, S5 and S6 Videos), these and the results of this study suggest this method to be a valuable alternative to existing protocol exploring foraging and operant behaviors in the honey bee.

These results suggest the strategies to access concealed food sources exist naturally, but experience can hasten the bee’s mastery of such strategies. Not only must bees solve complex discrimination problems, they must also solve mechanically challenging tasks in their daily routine [55, 56]. Indeed, honey bees have been observed exploiting flowers with unsuitable morphologies for pollinator resource collection such as plants with anemophilous characteristics [57]. Honey bees have also been observed manipulating papilionate flowers, such as *Robinia pseudoacacia*. These flowers have pollen release mechanisms for which the honey bee is often too physically weak to activate [58]. However, honey bees appear to learn to favor *R*. *pseudoacacia* flowers that are easier to trip and in many cases were able to trip the pollen release mechanism while accessing the nectar or finding a suitable foothold [57, 58]. Learning to manipulate flowers to access a nectar and pollen reward may be the natural foundations for motor-task operant behavior in honey bees.

We have previously argued [32, 59] that any behavior sensitive to response-reinforcer contingencies should not be automatically assumed to be an example of operant behavior. When the term operant behavior is applied to a particular invertebrate, the invertebrate should not only be able to manipulate an object but show that they know how to use it. The vertebrate literature is full of demonstrations in which an organism can be taught to press a lever at a particular speed, force, or directions. As far as we know there are no such demonstrations in the invertebrate literature. We believe the Cap Pushing Response method to be a prime candidate for continued exploration of operant behaviors in honey bees, and provides a potentially valuable comparative method for the functional analysis of behavior.

This method may also be of use for other psychological investigations in honey bees. The utilization of strategies to gain access to a concealed food source suggests honey bees are capable of utilizing knowledge of representation in regard to working memory tasks [60] which provides supporting evidence for ‘aboutness’ or ‘intentionality,’ a form of mental representation [61]. Representation in arthropods is an important point to consider in the discussion of consciousness [62]. However, when considering consciousness from different zoological levels, interpretations of behavior must consider the natural history of the animal [63–65].

## Conclusions

While honey bees are established as useful models to study intermediate levels of cognitive complexity and associated neural substrates [46, 66], our results provide further reason to investigate cognitive skills likened to advanced vertebrates. Relatively complicated foraging tasks where not all foragers solve the problem, or do so in different ways, are useful for examining the role of neurotransmitters in invertebrate decision processes [49]. What seemed like simple chance event mediated individual decision process development now appears to be influenced by slight differences in insect neurotransmitters [49]. Indeed, these techniques are advantageously suited for apicultural and ecological relevant studies such as investigating the effects of pesticides and pollutants on the foraging capabilities of honey bees [67]. Further, the learning-curve model fit demonstrated in this study provides a powerful tool for examining subtle differences in both the rate and degree of learning. Here we saw a pronounced difference in learning curves between auto- and explicit-shaped bees. Due to the ease of access to procedural materials, the cap pushing response provides a powerful tool for basic and applied research related to insect operant behavior and cognition.

## Supporting Information

S1 Text Methods for 3D Printed Materials. This document contains modified methodology for experiment procedure using 3D printed caps and feeding wells.
(DOCX)

S1 Video Explicit-Shaping. This bee is recruited to the food well and allowed to return five times before the cap is added (only the fifth visit shown in video). She is then given five trials of explicit-shaping through successive approximations. Following shaping, the well is completely covered and the bee pushes the cap to access the well. She becomes increasingly more proficient each visit. This video is cut, time-lapsed for length, and muted for file size. This file is for example purposes and should not be used for analysis. Different and complete videos are available upon request.
(MP4)

S2 Video Auto-Shaping. This bee is recruited to the food well and allowed to return five times before the cap is added (not shown in video). She is then given five trials of auto-shaping with a task of intermediate difficulty. Following auto-shaping, the well is completely covered and the bee pushes the cap to access the well. She becomes increasingly more proficient each visit. This video is cut, time-lapsed for length, and muted for file size. This file is for example purposes and should not be used for analysis. Different and complete videos are available upon request.
(MP4)

S3 Video Control Bees Example. Time-lapsed video of bees recruited to the food well but do not have experience pushing the cap.
(MP4)

S4 Video Error Example 1. Following five trials of successful cap pushing, a discrimination task was performed. The previously trained cap was moved to the side and a novel cap was used to cover the sucrose feeding well. The trained cap was counterbalanced between cross patterned and solid caps, and the novel cap presented for the discrimination task was different in shape. Upon first return, 11 of 12 bees pushed the cap previously trained, ignoring the cap that actually covered the sucrose well. If the bee pushed the cap off the plate, it was quickly returned. Bees would continue to push the incorrect cap when available (see S5 Video: Error Example 2).
(MP4)

S5 Video Error Example 2. Bees trained with one shape of cap (in this case cross patterned) repeatedly make the same error and push the previously trained cap. This is generalized to similar shaped caps as can be seen in the video.
(MP4)

S6 Video Error Example 3. This bee missed the well when pushing the cap and continued to push the cap for some time before reorienting to the sucrose well.
(MP4)
