# Supplementary material for: Operant Conditioning in Honey Bees (Apis mellifera L.): The Cap Pushing Response
Source: PLoS One. 2016 Sep 14;11(9):e0162347. doi: 10.1371/journal.pone.0162347 (PMC5023167; doi:10.1371/journal.pone.0162347)
Supplement: S1 Text — This document contains modified methodology for experiment procedure using 3D printed caps and feeding wells. (DOCX) [file pone.0162347.s001.docx]

**Video Resources and Extended Methods for 3D Printed Materials:**

The attached supplemental videos were taken at one of the Oklahoma State University’s Laboratory of Comparative Psychology and Behavioral biology apiaries. Using a laboratory hive and 3D printed materials, the studies presented were replicated and video-recorded. Slight variations to the methodology were required for use with the 3D printed procedural materials.

***3D Printed Cap and Plate Design.*** The feeding plate was 3D printed out of white acrylonitrile butadiene styrene (ABS) plastic using the Lulzbot Taz 4 3D printer (LulzBot.com; Aleph Objects, Inc.; Loveland, Colorado, USA). The plate was 15mm thick, with a diameter of 87mm. The feeding well was centered with an opening 6mm in diameter. The well was 6mm deep (spherical) from a 1mm thick overhang. The well held 20ml of sucrose solution. The solid circular cap used to cover the feeding well in test trials was 5mm thick and 14mm in diameter and weighed 0.49 grams. The cross patterned cap was 5mm thick from cross to base, 14mm in diameter, and weighed 0.50 grams. The cross pattern was raised 3mm from a 2mm base with crossbars measuring 3.6mm thick. The feeding plate was placed on a 12” by 18” by .5” thick green Stanton Trading cutting board positioned 50’ directly east from a laboratory hive.

***Recruitment.*** Forager honey bees were collected from a feeder with 25/75 (w/v) sucrose and water solution. Bees were moved from the sucrose feeder to the feeding plate via matchbox and allowed to feed on a 50/50 (w/v) sucrose solution in the feeding plate well. During feeding, these bees were marked with white Testors enamel paint. Marked bees were collected from the feeder and returned to the feeding plate until they returned of their own accord. Returning bees were then marked with a second color and allowed to return five times before shaping trials began. When a bee returned for the fifth time, all other bees were removed and the cap was introduced.

***Explicit-Shaping.*** The shaping procedure for the 3D printed caps differed from the original shaping method. The 3D printed caps were heavier and set flush on the surface providing no crevice or holes when completely covered. Thus, the shaping procedure focused on producing the required “pushing” response (see *Video 1: Explicit-Shaping*).

Following initial recruitment and pre-trial training, the well was covered almost completely, leaving a hole only large enough for the proboscis. The unshaped bee would utilize this hole to access the sucrose. To elicit the “pushing response,” the first trial of shaping involved a nearly covered feeding well that held enough sucrose for three visits from the honey bee. Given time, the bee would land and find the hole to feed. When the bee was done feeding and returned to the hive, the well was not refilled and the cap was returned to its original position if moved during feeding.

The second visit would often have considerably less latency to feed than the first visit. Following feeding, the well was not refilled but the cap was returned to its original position if moved. The third visit would require the honey bee to reach to the bottom of the sucrose well, inadvertently pushing the cap in the process. Following feeding, the well was then refilled to half capacity and the cap returned to its original position. The fourth and fifth visits followed the same procedure as the third visit but the access point was nearly closed allowing only a small sliver of access space.

***Auto-Shaping.*** Auto-Shaping trials did not have successive approximations, instead a task of intermediate difficulty was used. A cross-patterned cap was inverted (cross-pattern down) and placed over the well. Bees could access the sucrose well by pushing their proboscis through the opening provided (*Video 2: Auto-Shaping*). Five trials of auto-shaping were performed.

***Cap Pushing Response.*** Following five shaping trials, the cap was placed to completely cover the feeding well. This trial always resulted in a delay of well access, requiring, for the first time, the bee to push the cap to access the sucrose well. In response, the bee would repeatedly touch the cap with her proboscis, fly away and return, and explore the dish. This series of behavior would occur for roughly two to five minutes before the bee would attempt to push the cap. The cap pushing behavior varied slightly from one bee to the next, but the proboscis was primarily used to perform the act. All shaped bees eventually pressed the cap before the 10 minute cut-off. This protocol differs from the manuscript in that no control bees were able to press the cap before the 10 minute cut-off, all of which dropped out (see *Video 3: Control Bees Example*).

Five trials of cap pressing behavior were performed. Between each trial, the plate and cap were washed and sucrose replaced. The honey bee would display a reduced latency to push the cap with each visit. The following videos show the progression of the bee through the shaping and testing phases. A few notable and consistent errors appeared during informal manipulations. These error videos are included, but are informal observation; thus, they were not analyzed as additional components of the manuscript (See video files 4-6).
